# Increasing Human Performance by Sharing Cognitive Load Using Brain-to-Brain Interface

**DOI:** 10.3389/fnins.2018.00949

**Published:** 2018-12-13

**Authors:** Vladimir A. Maksimenko, Alexander E. Hramov, Nikita S. Frolov, Annika Lüttjohann, Vladimir O. Nedaivozov, Vadim V. Grubov, Anastasia E. Runnova, Vladimir V. Makarov, Jürgen Kurths, Alexander N. Pisarchik

**Affiliations:** ^1^REC “Artificial Intelligence Systems and Neurotechnology”, Yuri Gagarin State Technical University of Saratov, Saratov, Russia; ^2^Institute of Physiology I, University of Münster, Münster, Germany; ^3^Potsdam Institute for Climate Impact Research, Potsdam, Germany; ^4^Department of Physics, Humboldt University, Berlin, Germany; ^5^Institute for Complex Systems and Mathematical Biology, University of Aberdeen, Aberdeen, United Kingdom; ^6^Center for Biomedical Technology, Technical University of Madrid, Madrid, Spain

**Keywords:** brain-computer interface (BCI), brain-to-brain interface (BBI), human-to-human interaction, visual attention, brain states recognition, cognitive performance, cognitive reserve, mental fatigue

## Abstract

Brain-computer interfaces (BCIs) attract a lot of attention because of their ability to improve the brain's efficiency in performing complex tasks using a computer. Furthermore, BCIs can increase human's performance not only due to human-machine interactions, but also thanks to an optimal distribution of cognitive load among all members of a group working on a common task, i.e., due to human-human interaction. The latter is of particular importance when sustained attention and alertness are required. In every day practice, this is a common occurrence, for example, among office workers, pilots of a military or a civil aircraft, power plant operators, etc. Their routinely work includes continuous monitoring of instrument readings and implies a heavy cognitive load due to processing large amounts of visual information. In this paper, we propose a brain-to-brain interface (BBI) which estimates brain states of every participant and distributes a cognitive load among all members of the group accomplishing together a common task. The BBI allows sharing the whole workload between all participants depending on their current cognitive performance estimated from their electrical brain activity. We show that the team efficiency can be increased due to redistribution of the work between participants so that the most difficult workload falls on the operator who exhibits maximum performance. Finally, we demonstrate that the human-to-human interaction is more efficient in the presence of a certain delay determined by brain rhythms. The obtained results are promising for the development of a new generation of communication systems based on neurophysiological brain activity of interacting people. Such BBIs will distribute a common task between all group members according to their individual physical conditions.

## 1. Introduction

The brain-computer interface (BCI) development is a novel multidisciplinary research topic in neuroscience, physics and engineering. Many applications, including medicine, industry, robotics, etc. (Chen et al., 2015; Bowsher et al., 2016; Kawase et al., 2017; Spüler, 2017; Maksimenko et al., 2017a) are in dire need of this modern technology. The BCI is based on the characteristic forms of electrical or magnetic brain activity and their real-time transformation into computer commands. Today, the developed neuro-computer interfaces allow controlling a cursor 2D movement (Wolpaw and McFarland, 2004, partially synthesize speech Birbaumer et al., 1999, and simplest human movements Ma et al., 2017). The BCIs can be effectively used in neuroprosthetics Ma et al. (2017), rehabilitation Daly and Wolpaw (2008), exoskeletons Kawase et al. (2017), and robots Peternel et al. (2016). In addition, recent advances in cognitive neuroscience provide the possibility of using BCIs for enhancing cognitive abilities and treating mental disorders (Hillard et al., 2013). Moreover, BCIs are expected to enhance human performance, such as to shorten reaction time, to improve error processing and unsupervised learning (Mirabella and Lebedev, 2016), etc.

Since the main goal of BCI is to repair and/or increase human performance in solving different tasks (Zander and Kothe, 2011; Chaudhary et al., 2016), the machine controlled by the brain activity takes a part of the cognitive or physical human load. Similarly to the human-machine interaction, a human-to-human interaction can be improved by enhancing human collaboration with the help of BCI. In this situation, the machine component of traditional BCI can be replaced by another human linked to the first one by an interface, whose assistance would enhance the subject performance in managing a particular task. Such a brain-to-brain interface (BBI) would be very helpful for a group of people subjected to a common task which requires sustained attention. In everyday practice, this is a common occurrence, for example, among office workers, pilots of military (Estrada et al., 2012) or civil aircrafts (Sallinen et al., 2017), power plant operators, and other teams, whose routine work includes continuous monitoring of instrument readings, and requires sustained alertness and concentration (Baker et al., 1990; Jensen, 1999; Takahashi et al., 2005). The human-to-human interaction through computers could help the members of such groups to effectively interact by estimating and monitoring physical conditions of each person, in particular, degree of alertness, in order to distribute workloads among all participants according to their current physiological status.

**Figure 1:**
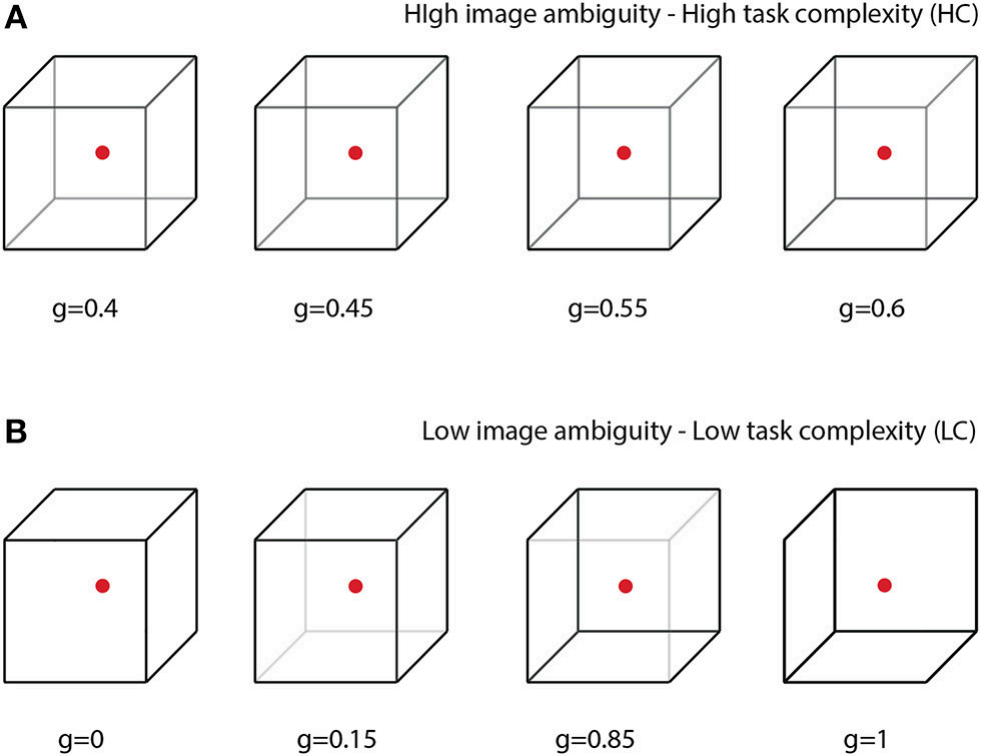
Complete set of visual stimuli split into two sets: **(A)** cubes with high degree of ambiguity representing tasks of high complexity (HC) and **(B)** cubes with low degree of ambiguity representing tasks of low complexity (LC).

In this paper, we propose a special BBI to heighten human-to-human interaction while performing a common collective task. The efficiency of the proposed BBI is estimated in experimental sessions, where the participants perform a prolonged task of classification of bistable visual stimuli of different degree of ambiguity. In bistable perception, the ambiguity is related to the probability of different interpretations of the presented image; highest ambiguity results in about 50% probability. In our experiment, we explore a classical example of visual ambiguous stimuli, the Necker cube (see Figure 1). This bistable image is very convenient for research, because unlike other ambiguous images, its ambiguity can be adjusted by varying the control parameter *g*∈[0, 1] related to the contrast of the inner ribs, which define the volume structure of this 2D image. The values *g* = 0 and *g* = 1 correspond to unambiguous left- and right-oriented cubes, respectively. The parameter *g* controls the probability of the cube orientation interpretation. For instance, the probability of the left-oriented cube interpretation is 100% when *g* = 0 and 0% when *g* = 1. We assume that the complexity of the cognitive task in the cube classification as left or right oriented one is closely related to the cube ambiguity. Evidently, the stimuli with low ambiguity can be classified easier than the stimuli with high ambiguity (see section 2.). Therefore, the classification of unambiguous or weakly ambiguous images is considered as a low-complexity (LC) task, whereas the classification of highly ambiguous images as a high-complexity (HC) task.

## 2. Materials and Methods

### 2.1. Participants

Twenty healthy unpaid volunteers, 12 males and 8 females, between the ages of 20 and 43 with normal or corrected-to-normal visual acuity participated in the experiment. All of them provided informed written consent before participating. The experimental studies were performed in accordance with the Declaration of Helsinki and approved by the local research Ethics Committee of the Yuri Gagarin State Technical University of Saratov. The participants did not exhibit substantial differences in factors characterizing properties of attention according to the results of psychodiagnostic tests (Maksimenko et al., 2018).

### 2.2. Visual Task

All participants were subjected to the visual task of classification of consistently presented Necker cubes as left- or right-oriented (Figure 1). The Necker cube (Necker Esq, 1832) is a popular object of many psychological experiments (Mathes et al., 2006; Kornmeier et al., 2011; Pisarchik et al., 2014). An observer without any perceptional abnormalities perceives the Necker cube as a bistable 3D-object due to the specific position of the cube's ribs. Bistability in perception consists in interpretation of the cube orientation, depending on the contrasts of the inner ribs. To control the cube ambiguity, we used the normalized value *g* = *y*/255 ∈ [0, 1], where *y* is the brightness of three inner lines centered in the left middle corner (according to the 8-bit grayscale palette) (Runnova et al., 2016; Hramov et al., 2017). The values *g* = 1 and *g* = 0 correspond, respectively, to 0 (black) and 255 (white) pixels' luminance of these lines. The Necker cube images with different *g* were created using a standard graphics software. One can see that the parameter *g* controls the cube orientation. Along with samples of unambiguous left- (*g* = 0) or right- (*g* = 1) oriented cubes, as well as a fully ambiguous cube (*g* = 0.5), we successively presented cubes of various ambiguity with (0 < *g* < 0.5) and (0.5 < *g* < 1), each of which differed from the previous one.

The value of *g* was considered as a *degree of complexity* in the cube classification, because the cubes with *g* close to 1 or 0 can easily be interpreted as left- or right-oriented, respectively, whereas the interpretation of the cubes with *g* close to 0.5 is a more complex task. According to the difficulty of the classification task, the whole set of the presented stimuli with *g* = (0, 0.15, 0.4, 0.45, 0.55, 0.6, 0.85, 1) was split into two subsets: the task of high complexity (HC) to classify highly ambiguous images with *g* = (0.4, 0.45, 0.55, 0.6) (Figure 1A) and the task of low complexity (LC) to classify weakly ambiguous cubes with *g* = (0, 0.15, 0.85, 1) (see Figure 1B). The subjects were asked to classify the cubes as left/right oriented according to their first impression.

### 2.3. Experimental Procedure

All participants were instructed to press either left or right key depending on their first impression on the cube orientation at each presentation, the left key when they saw the left-oriented cube and the right key otherwise. The subjects were randomly divided into 10 pairs. Each pair was subjected to two different experiments: EXP1 and EXP2, both containing two sessions: session 1 (S1) and session 2 (S2), each lasted 30 min. In S1, the cubes with randomly selected *g* were simultaneously presented to both subjects in each pair; every set of stimuli was presented for about 30 times. In S2, the whole set of stimuli was split into two subsets: stimuli with high ambiguity (HC) (see Figure 1A) and stimuli with low ambiguity (LC) (see Figure 1B), and cubes from different subsets were presented depending on brain responses of the participants.

- EXP1: S1–both subjects simultaneously observed the same cubes, randomly selected from a whole set of stimuli; S2–task complexity was distributed among participants based on their instantaneous alertness; the subject with higher alertness received a HC stimulus, while his/her partner received a LC stimulus. S2 was associated with human-human interaction through a non-delayed coupling, i.e., task complexity was distributed based on instantaneous alertness of the participants.
- EXP2: S1–both subjects simultaneously observed the same cubes, randomly selected from a whole set of stimuli (the same as in EXP1); S2–the subject with higher alertness received HC stimuli, while his/her partner received LC stimuli only in the case when the difference between their degrees of alertness became >10%. S2 was associated with delayed coupling between the participants because the task was not switched immediately.

In addition, we carried out a third experiment, where subjects were not arranged in pairs, but each performed the individual classification task. This additional experiment also consisted of two sessions, each lasted 30 min, during which the participant observed low ambiguous stimuli (in the first session) and high ambiguous stimuli (in the second session). Other parameters of the experiment are shown in Table 1.

**Table 1:** Parameters of the experiment.

**Parameter**	**Value**
Time interval of visual stimuli presentation	randomly chosen between 1 and 1.5 s
Time interval between visual stimuli presentations	randomly chosen between 3 and 5 s
Number of presented visual stimuli	200
Total duration of experimental session	30 min
Location of EEG scalp electrodes	International 10–20 system
EEG recording sampling rate	250 Hz
EEG recording filtering	1–30 Hz
Considered EEG channels	*O*_1_, *O*_2_, *P*_3_, *P*_4_, *P*_*z*_
Considered EEG bands	α-waves (8–12 Hz), β-waves (15–30 Hz)

### 2.4. EEG Recording

To record EEG data, we used cup adhesive Ag/AgCl electrodes placed on the “Tien–20” paste. Immediately before the experiments started, we performed all necessary procedures to increase the conductivity of the skin and reduce its resistance using abrasive “NuPrep” gel. The impedance values were measured after the electrodes were installed, and monitored during the experiments. Usually, the impedance varied in the interval of 2–5 kΩ. The ground electrode *N* was located in front of the head at the Fpz electrode location. The EEG signals were filtered by a band-pass filter with cut-off points at 1 Hz (HP), both 100-Hz (LP) and 50-Hz Notch filters. The electroencephalograph “Encephalan-EEGR-19/26” (Medicom MTD company, Taganrog, Russian Federation) with multiple EEG channels and two-button input device (keypad) was used for amplification and analog-to-digital conversion of the EEG signals. This device possessed the registration certificate of the Federal Service for Supervision in Health Care No. FCP 2007/00124 of 07.11.2014 and the European Certificate CE 538571 of the British Standards Institute (BSI).

To diminish artifacts of the muscular origin, we asked the participants to take a pose which excluded excessive tension of neck muscles. Recent studies showed that these artifacts affect time frequency properties in occipital area in the spectral bandwidth of muscle activity (~20–300 Hz) (Muthukumaraswamy, 2013). It is slightly overlapped with the frequency band of 1–30 Hz explored in the present work. On the other hand, the amplitude of muscular artifacts in the occipital area was strongly related to the subject's posture during experiment. Since we only estimated relative changes in spectral properties during time intervals when the stimuli were presented with respect to the intervals when the subject did not receive the stimuli, the effect of muscular artifacts uncorrelated with the stimuli presentation was not strong.

### 2.5. Estimation of the Brain Response

The perception of visual stimuli is known to be associated with an increase in the electrical activity in visual areas in the occipital lobe (Mulckhuyse, 2011; Gleiss and Kayser, 2014) and attentional areas in the parietal lobe (Laufs, 2006). Therefore, the most informative channels are in these lobes. This allowed us to restrict our analysis to the EEG recordings from five electrodes only (O_1_, O_2_, P_3_, P_4_, P_*z*_), located in the occipital (O1 and O2) and parietal (P3, P4, Pz) lobes according to the 10–20 electrode layout (Niedermeyer and da Silva, 2014), to be able to perform fast analysis in real time.

The EEG data was analyzed using the continuous wavelet transform (Pavlov et al., 2012). The wavelet energy spectrum $E^{n}\left(f,t\right)=\sqrt{W_{n}{\left(f,t\right)}^{2}}$ was calculated for each EEG channel *X*_*n*_(*t*) in the *f* ∈ [1, 30]-Hz frequency range. Here, *W*_*n*_(*f, t*) is the complex-valued wavelet coefficients calculated as

(1) $$\begin{array}{l} W_{n}\left(f,t\right)=\sqrt{f}\overset{t+4/f}{\underset{t-4/f}{\int }}X_{n}\left(t\right)\psi^{*}\left(f,t\right)dt, \end{array}$$

where *n* = 1, …, *N* is the EEG channel number (*N* = 5 being the total number of channels used for the analysis) and “*” defines the complex conjugation. The mother wavelet function ψ(*f, t*) is the Morlet wavelet often used for the analysis of neurophysiological data, defined as

(2) $$\begin{array}{l} \psi \left(f,t\right)=\sqrt{f}\pi^{1/4}{\text{e}}^{j\omega_{0}f\left(t-t_{0}\right)}{\text{e}}^{f{\left(t-t_{0}\right)}^{2}/2}, \end{array}$$

where ω_0_ = 2π is the central frequency of the Morlet mother wavelet (Sitnikova et al., 2014).

**Figure 2:**
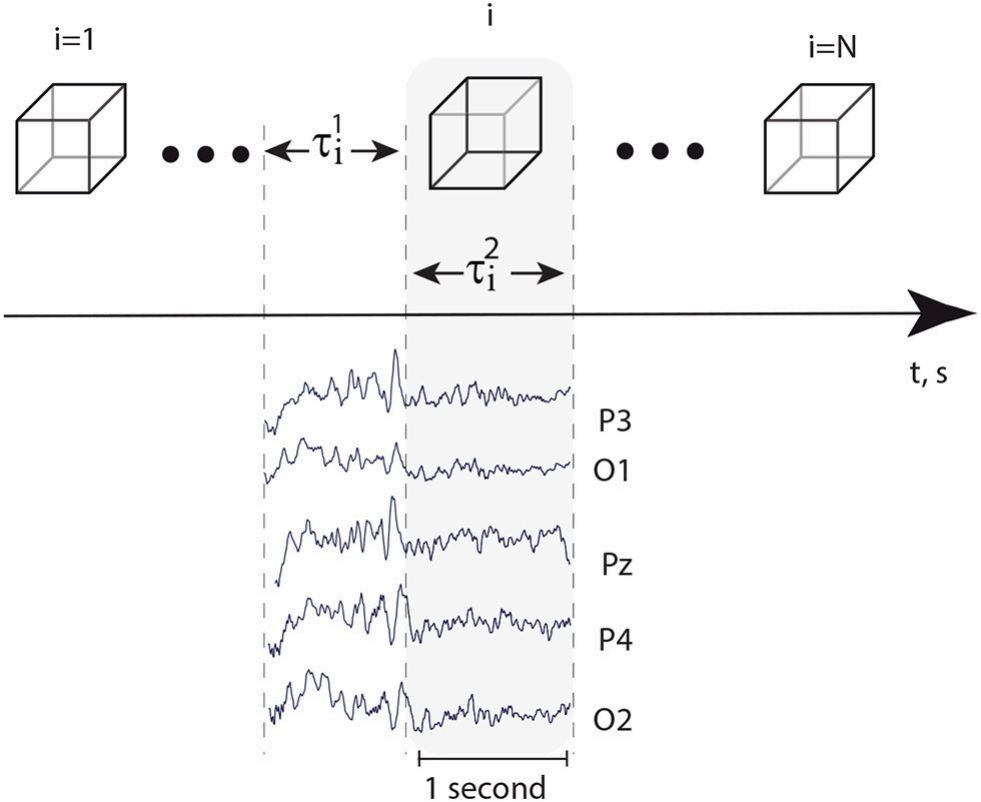
**Top:** successively presented visual stimuli and time intervals $\tau_{i}^{1}=1$ s and $\tau_{i}^{2}=1$ s, preceded stimuli presentation and followed immediately by the moment of stimulus presentation, respectively. **Bottom:** typical EEG traces registered in occipital area during time intervals $\tau_{i}^{1}$ and $\tau_{i}^{2}$.

Every EEG signal associated with the presentation of a single visual stimulus was analyzed separately in the alpha and beta frequency bands during a 1-s interval preceding the presentation ($\tau_{i}^{1}$) and a 1-s interval followed by the moment of the stimulus presentation ($\tau_{i}^{2}$) (Figure 2). A special software controlled digital triggering to initiate the calculation process together with stimulus presentation.

As a result, the values $A_{i}^{1}$, $A_{i}^{2}$, $B_{i}^{1}$, $B_{i}^{2}$ were calculated for each *i*-th presentation as

(3) $$\begin{array}{l} A_{i}^{1,2}=\overset{N}{\underset{n=1}{\sum }}\int_{t\in \tau_{i}^{1,2}}\xi^{n}\left(t^{\prime }\right)dt^{\prime }, \end{array}$$

(4) $$\begin{array}{l} B_{i}^{1,2}=\overset{N}{\underset{n=1}{\sum }}\int_{t\in \tau_{i}^{1,2}}\xi^{n}\left(t^{\prime }\right)dt^{\prime }, \end{array}$$

where

(5) $$\;\xi^{n}(t)=\{\begin{array}{lll} 1, & \text{if} & f_{max}^{n}\in \Delta f_{\alpha ,\beta },\\ 0, & \text{if} & f_{max}^{n}\notin \Delta f_{\alpha ,\beta }, \end{array}$$

where *N* = 5 is the number of EEG channels and $f_{max}^{n}$ is the location of the maximal spectral component. The values given by Equations (3, 4) quantify neural activity in α- and β-frequency bands. According to the recent work (Maksimenko et al., 2018), visual attention is related to the interplay between these bands in occipital and parietal areas. In particular, changes in α-activity are associated with visual (Sauseng et al., 2005) or auditory attention (Foxe and Snyder, 2011), while changes in β-activity are associated with stimuli processing (Sehatpour et al., 2008) and switching the brain to an attention state (Wróbel, 2000; Gola et al., 2013). The role of alpha and beta activity in the perceptual process is also highlighted in Michalareas et al. (2016) in the context of information transfer in visual areas.

The control characteristic *I*(*i*) was calculated as follows

(6) $$\begin{array}{l} I\left(i\right)=\frac{\left(a_{i}^{1}-a_{i}^{2}\right)-\left(b_{i}^{2}-b_{i}^{1}\right)}{2}, \end{array}$$

where $a_{i}^{1,2}$ and $b_{i}^{1,2}$ were obtained as

(7) $$\begin{array}{l} a_{i}^{1,2}=\frac{1}{6}\overset{i}{\underset{n=i-6}{\sum }}A_{n}^{1,2}, \end{array}$$

(8) $$\begin{array}{l} b_{i}^{1,2}=\frac{1}{6}\overset{i}{\underset{n=i-6}{\sum }}B_{n}^{1,2} \end{array}$$

by averaging $A_{i}^{1,2}$ and $B_{i}^{1,2}$ values over six presentations.

The value of *I*(*i*) calculated in real time using Equation (6) reflects the intensity of the brain response on the appearing visual stimuli. Large *I*(*i*) is associated with a high response due to more careful image processing by the subject, whereas small *I*(*i*) is related to a low response, which takes place when the subject does not pay much attention on the classification task.

The design of our algorithm allowed us to take into account changes in the time-frequency EEG structure associated with the stimulus perception and neglect the influence of unrelated oscillations, e.g., muscular artifacts which occurred in the EEG signals at the moments uncorrelated with the stimuli presentation.

### 2.6. Brain-to-Brain Interface (BBI)

**Figure 3:**
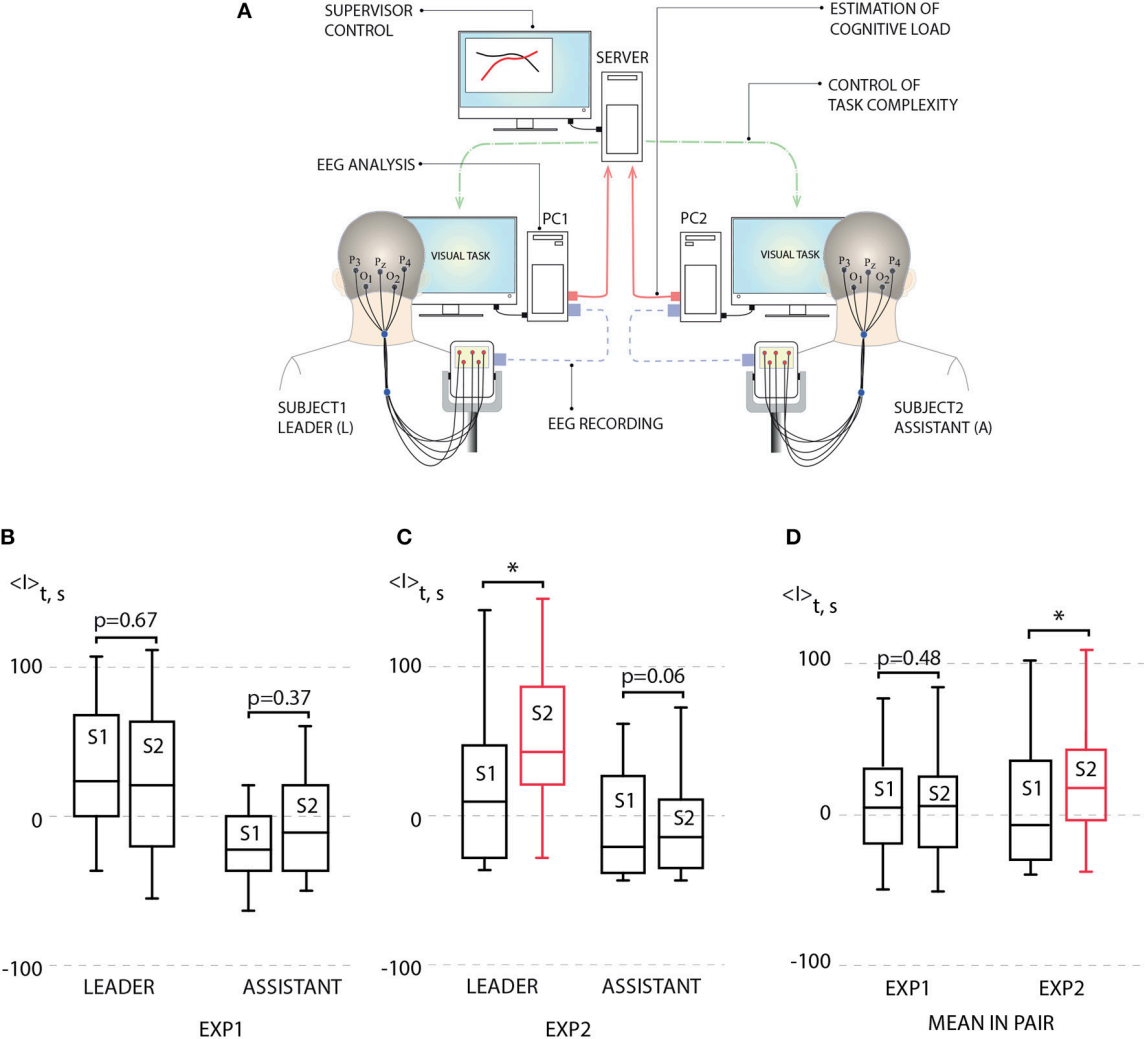
**(A)** Schematic illustration of human-to-human interaction through BBI. **(B)** Left: average leader's brain response 〈*I*〉 during first experiment (EXP1): session 1 (S1) [no link between subjects, *p* = 0.938 by Shapiro-Wilk normality test (SWNT)] and session 2 (S2) (no delay in coupling between subjects, *p* = 0.965 by SWNT) (not significant, *n* = 10, *p* = 0.67 by paired sample *t*-test); right: average assistant's brain response 〈*I*〉 during EXP1: S1 (no link between subjects, *p* = 0.402 by SWNT) and S2 (no delay in coupling between subjects, *p* = 0.485 by SWNT) (not significant, *n* = 10, *p* = 0.37 by paired sample *t*-test). **(C)** Left: average leader's brain response 〈*I*〉 during second experiment (EXP2): S1 (no link between subjects, *p* = 0.131 by SWNT) and S2 (delayed coupling between subjects, *p* = 0.889 by SWNT) (significant change, *n* = 10, ^*^p < 0.05 by paired sample t-test); right: average assistant's brain response 〈*I*〉 during EXP2: S1 (no link between subjects, *p* = 0.099 by SWNT) and S2 (delayed coupling between subjects, *p* = 0.169 by SWNT) (not significant, *n* = 10, *p* = 0.06 by paired sample t-test). **(D)** Left: mean brain response 〈*I*〉 for pairs during EXP1: S1 (no link between subjects, *p* = 0.979 by SWNT) and S2 (no delay in coupling between subjects, *p* = 0.847 by SWNT) (not significant, *n* = 10, *p* = 0.48 by paired sample t-test); right: mean brain response 〈*I*〉 for pairs during EXP1: S1 (no link between subjects, *p* = 0.108 by SWNT) and S2 (no delay in coupling between subjects, *p* = 0.622 by SWNT) (significant change, *n* = 10, ^*^p < 0.05 by paired sample *t*-test). Medians (bars), 25–75 percentiles (box) and outlines (whiskers) are shown.

The scheme of the proposed BBI is illustrated in Figure 3A. The BBI performs human-to-human interaction in the following way:

- The stimulus (Necker cube) is simultaneously presented to a pair of operators (subject 1 and subject 2) on the corresponding client personal computers (PC1 for subject 1 and PC2 for subject 2). Each subject is able to see only his/her screen, but not the screen of another subject.
- Subjects' EEGs are simultaneously recorded and transmitted in real time to the corresponding PCs. The operator's performance is estimated using stimulus-related brain response *I*(*i*) to every presented *i*-th stimulus on the base of the EEG spectral properties (see Methods).
- Brain responses *I*_1_(*i*) and *I*_2_(*i*) of subject 1 and subject 2, respectively, are transmitted to the computational server for a comparative analysis.
- Depending on the result in this comparison, the corresponding control command is sent to each PC to adjust the ambiguity range of the presented stimuli for each subject. For example, if *I*_1_(*i*)>*I*_2_(*i*), then subject 1 receives a stimulus with higher ambiguity, while subject 2 receives a stimulus with weaker ambiguity.

The BBI was built based on the “Encephalan-EEGR-19/26” (Medicom MTD, Russia). The EEG data recorded from each subject were initially processed by local computers, where the values of the brain response were calculated individually for each subject using home-made software based on C++. Then, these values were sent to a calculation server with markers containing the information about presented stimuli through the IP-message. The server software (also developed in C++) analyzed the incoming information and sent control commands back to the local computers through IP-messages. The control commands were used to realize switches between the sets of stimuli. The information about the set number was sent back to the server. As a result, the dataset containing the values of brain responses and the stimuli set numbers were saved to a data file at every time moment.

### 2.7. Connectivity Analysis

The connectivity analysis for every pair of participants was performed via the Recurrence-based Measure of Dependence (RMD) proposed and described in details in Goswami et al. (2013). Using this approach, we could either identify coupling directions and time lags between interacting systems or prove that they were independent.

Let *I*_1_(*i*) and *I*_2_(*i*) be L (leader) and A (assistant) brain response time series, respectively. The RMD is calculated as

$$\text{RMD}\left(\tau \right)=\log_{2}\left(\frac{1}{N^{\prime }}\sum_{i=1}^{N^{\prime }}{\text{RMD}}_{i}\left(\tau \right)\right),$$

$${\text{RMD}}_{i}\left(\tau \right)=\frac{P\left(I_{1}\left(i\right),I_{2}\left(i+\tau \right)\right)}{P\left(I_{1}\left(i\right)\right)P\left(I_{2}\left(i+\tau \right)\right)},$$

where τ is a time lag, *P*(*I*_*k*_ = *I*_*k*_(*i*)) is a probability for *I*_*k*_ to take the value *I*_*k*_(*i*), and *P*(*I*_1_(*i*), *I*_2_(*i*)) = *P*(*I*_1_ = *I*_1_(*i*)) *P*(*I*_2_ = *I*_2_(*i*)) is a joint probability that *I*_1_ = *I*_1_(*i*) at the same time, where *I*_2_ = *I*_2_(*i*) and *N*′ = *N*−τ. The probabilities *P* determined by a recurrence matrix (Marwan et al., 2007), is calculated as

$$P\left(I_{k}\left(i\right)\right)=\frac{1}{N}\sum_{j=1}^{N}R_{k}\left(i,j\right),$$

$$P\left(I_{1}\left(i\right),I_{2}\left(i\right)\right)=\frac{1}{N}\sum_{j=1}^{N}JR\left(i,j\right),$$

$$JR=R_{1}\left(i,j\right)R_{2}\left(i,j\right),$$

where **R**_*k*_(*i, j*) is a recurrence matrix of *k*-th participant and **JR**(*i, j*) is a joint recurrence matrix.

Next, we carried out the statistical test of significance of the obtained RMD(τ) values using surrogate data of the observed time series. For this aim, we generated so-called twin surrogates, which were independent realizations of the entire system via the recurrence-based approach proposed in Thiel et al. (2006) and Ramos et al. (2017). The observed values of RMD(τ) implied a statistically significant dependence of *I*_2_ on *I*_1_ if RMD exceeded 95 percentile (confidence interval) of the test RMD(τ) distribution calculated for *I*_1_ surrogate time series. In the framework of this approach, we found that for τ > 0 a non-zero RMD exceeding the confidence interval determined the dependence of *I*_2_ on *I*_1_, and the converse was true for τ < 0. On the contrary, if RMD lied inside the confidence interval, the participants acted independently.

### 2.8. Statistical Analysis

The statistical analysis was performed using IBM SPSS Statistics. The values of the brain response *I*(*t*) calculated during different sessions (or for different participants) were compared as two independent samples using an independent samples *t*-test. The mean values of the brain response *I*(*t*) within the group of participants were compared for different experimental sessions as paired samples. First, we applied the Shapiro-Wilk normality test to the corresponding samples. If the data set did not pass this test (*p* < 0.05), we applied the Wilcoxon signed rank test, otherwise, *t*-test was used. The tests used for the statistical analysis are indicated in the caption of Figure 3.

## 3. Results

For each session, average performance 〈*I*〉 was calculated for each subject by averaging his/her brain response *I* over 200 image presentations. According to 〈*I*〉 estimated during preliminary non-coupled session (S1), the subjects in each pair were classified as a leader (L) (subject with higher 〈*I*〉) and an assistant (A) (subject with lower 〈*I*〉). Then, 〈*I*〉 of L and A obtained during uncoupled and coupled sessions were calculated and compared.

The results of this comparison for EXP1 are presented in Figure 3B in the form of box-and-whiskers diagrams which show average performance 〈*I*〉 for leaders and assistants in all pairs. One can see that according to the group analysis, the interaction between subjects in EXP1 did not bring a significant effect on the degree of performance for leaders and assistants. On the contrary, we uncovered a significant increase in the degree of alertness of the leader in EXP2 shown in Figure 3C, where the task complexity was changed if a 10% difference appeared between values of *I*_1_(*i*) and *I*_2_(*i*). The observed changes in the assistants' degree of alertness were insignificant. Such an increase in the leader's performance caused an enhancement of the pair's performance. This can be seen from Figure 3D, where we plot the value of 〈*I*〉 averaged over all participants' performances.

**Figure 4:**
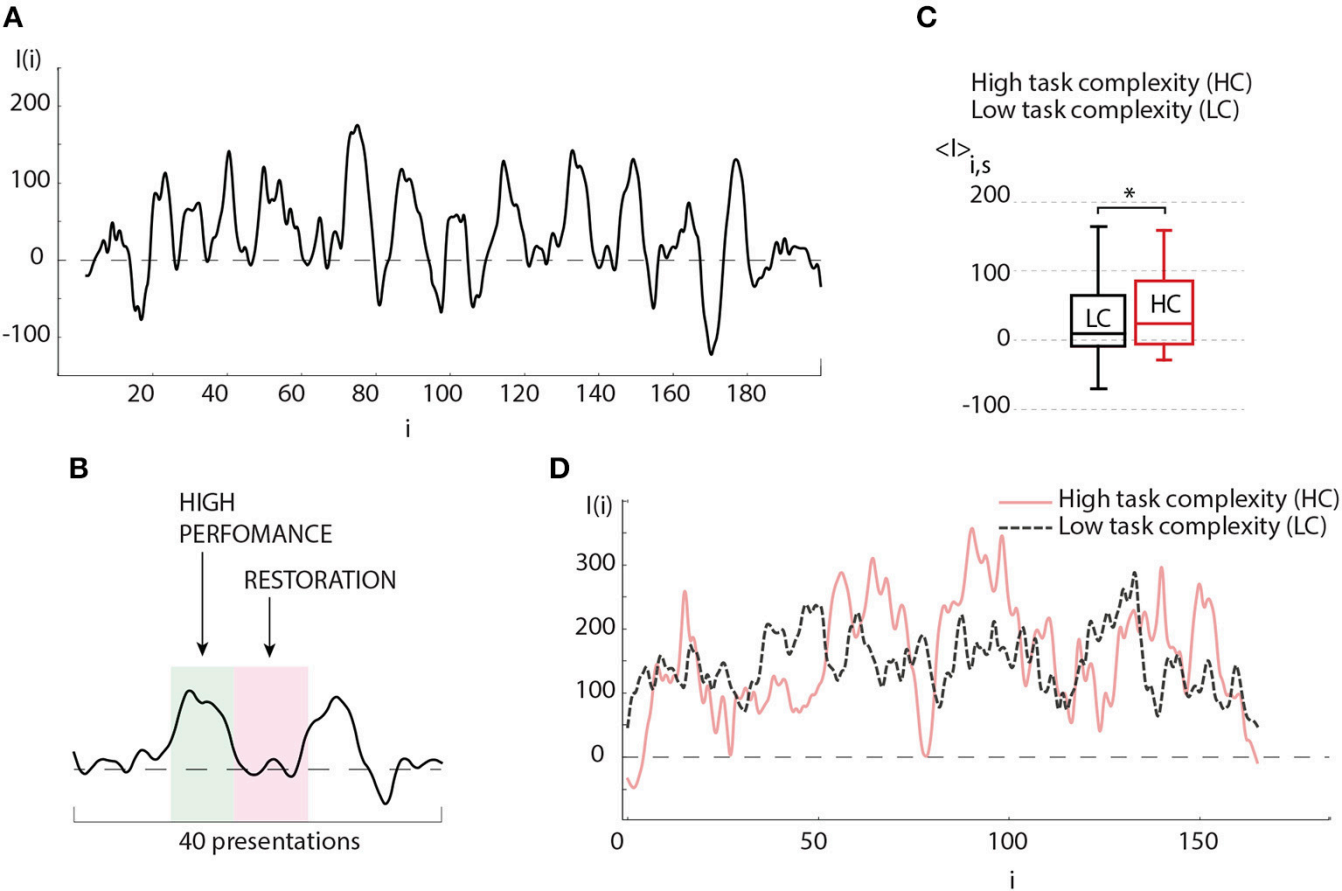
**(A)** Typical oscillations of the brain response value (*I*) depending on the number of presented visual stimuli during the experimental session. **(B)** Detailed illustration of a single period of (*I*) oscillations (≈20 presented stimuli), where “hump” and “hollow” demonstrate the states of high brain performance and restoration, respectively. **(C)** Average brain response 〈*I*〉 of the subject during experimental session, when images with low degree of ambiguity (LC) (*p* = 0.436 by Shapiro-Wilk normality test) and high degree of ambiguity (HC) (*p* = 0.434 by Shapiro-Wilk normality test) were presented (session contains 200 presentations) (significant change, *n* = 10, ^*^p < 0.05 by paired sample *t*-test). Medians (bars), 25–75 percentiles (box) and outlines (whiskers) are shown. **(D)** Typical oscillations of the brain response value (*I*) depending on the number of presented visual stimuli during the perception of images of high degree (solid curve) and low degree (dashed curve) of ambiguity.

In order to understand the obtained result, let us now consider the evolution of the brain response during one experimental session. The typical dependence *I*(*i*) reflects a change in the amplitude of the brain response as the number of presented Necker cubes *i* is increased. The result shows oscillations whose period varies from 15 to 40 presented stimuli (see Figure 4A). Such a behavior of the brain response can be associated with the relaxation of the neural ensemble. The restoration state is needed for cognitive recovery after mental fatigue caused by the HC task (Figure 4B). The degree of image ambiguity (or complexity of the visual task) strongly affects the amplitude of the brain response. In Figure 4C we plot the average values of the brain response calculated for the preliminary experimental session conducted individually for each participant, where visual stimuli with low degree of ambiguity were presented. The base value was chosen when the observer was subjected to highly ambiguous stimuli. In Figure 4D typical dependence I(i) are compared for LC and HC tasks. We found that an increase in the degree of image ambiguity (or an increase in the task complexity) leads to a corresponding increase in the average amplitude of the brain response 〈*I*〉. According to our recent study (Maksimenko et al., 2017b), such a change in the brain response is caused by an increase in alertness. In this case, the time-frequency structure of the EEG signals exhibits significant changes for each subsequent *i*-th stimulus. The origin of these changes is in the contribution of α and β brain rhythms. From the viewpoint of neural dynamics, this means that a large neural population participates in image classification (Maksimenko et al., 2017c). As the image ambiguity grows, the average response *I*(*i*) increases.

According to the above result, one can assume that effective classification can be obtained if the image ambiguity is adjusted according to the current value of the brain response. This can be implemented with the help of biological feedback according to the following situations: (i) in the case of high operator performance defined by a high value of the brain response base line, the operator is subjected to stimuli with high degree of ambiguity, i.e., the task complexity is being increased with corresponding cognitive load increment, (ii) as the cognitive load is increased, it leads to an augmentation in neuronal tiredness which immediately causes a decrease in the brain response, (iii) in contrast, having a low brain response the operator is subjected to stimuli with a low degree of ambiguity, i.e., the cognitive load decreases, and (iv) a decrease in cognitive load causes a faster restoration.

In the case of two operators, each of them was subjected to the same stimuli ambiguity cycle in antiphase. This design offers two main advantages: (i) the most complicated (or most important) task is being processed by the operator with highest momentary skill, (ii) the switch to the less complicated task, when the operator is tired, allows decreasing the restoration time. The combination of both features enables to efficiently manage the whole workload distribution. Obviously, the efficiency of the described approach depends on the coincidence quality between complexity and operator's performance skills, namely, high performance has to be correlated with high complexity, and the restoration phase should be correlated with low complexity defined by the frequency of *I*(*i*) oscillations.

**Figure 5:**
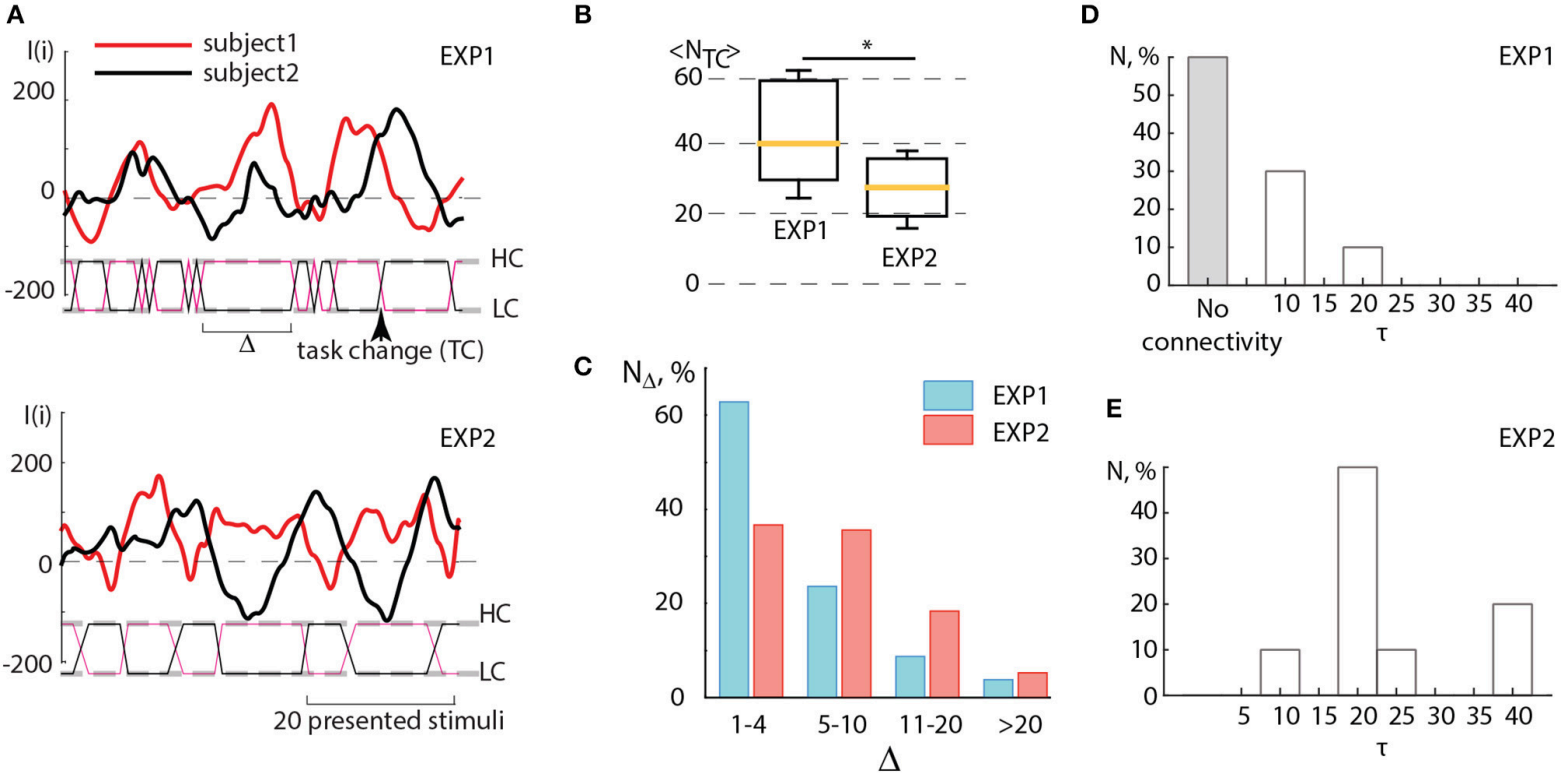
**(A)** Detailed illustration of the BBI performance during experiments EXP1 and EXP2. Solid curves represent the brain response *I*(*i*) of two partners, dashed lines marked as HC and LC indicate two types of visual tasks associated with high and low complexity, solid lines between these dashed lines indicate switches (TC) between the tasks, the arrow shows the moment of a single switch. The length of the interval (Δ) between two successive switches is measured in the number of presented stimuli. **(B)** Comparison between the number of switches < *N*_*TC*_ > averaged over subjects during experiments EXP1 and EXP2. Statistical significance is estimated via paired *t*-test. Medians (yellow bars), 25–75 percentiles (box) and outlines (whiskers) are shown. **(C)** Distribution of time intervals between switches Δ calculated by taking into account four ranges: rapid switches (1–4 units), medium switches (5–10 units), optimal switches (11–20 units) and long switches (>20 units). **(D,E)** Connectivity analysis. Histograms reflecting distribution of coupling time lags τ over all pairs of participants showing the change in the character of coupling in **(D)** EXP1 and **(E)** EXP2. ^*^p < 0.05 by paired sample *t*-test.

In order to check how this criterium of coincidence was satisfied in two conducted experiments, EXP1 and EXP2, we carried out the detailed analysis of the results obtained in the corresponding experimental sessions. When comparing the sub-plots in Figure 5A, one can clearly see that during EXP1, when the task complexity switches immediately as soon as the amplitude of the brain response of one subject (*I*_1_(*i*)) exceeds the brain response of the other subject (*I*_2_(*i*)), there are many short switches with Δ < 5, smaller than the period of *I*(*i*) oscillations. In this case, the dependencies *I*_1_(*i*) and *I*_2_(*i*) obtained for both subjects do not demonstrate an antiphase mode. On the contrary, the values of *I*(*i*) obtained for the subjects during experiment EXP2 behave mostly in antiphase and switches appear less frequently. In Figure 5B the box-and-whiskers diagram compares the mean number of switches 〈*N*_*TC*_〉 averaged over subjects during EXP1 and EXP2. One can see that in EXP2 the number of switches significantly decreases (significance is judged from *p* < 0.05 estimated via paired t-test).

One can surmise that in EXP1 the multiple unnecessary spontaneous switches, caused by low-frequency fluctuations of *I*(*i*), interfered with the establishment of the antiphase mode between oscillations *I*_1_ and *I*_2_ of the leader and assistant in pair. Unlike experiment EXP1, during EXP2 such switches appeared more scarcely and the interval Δ between two successive switches matched the period of *I*(*i*) oscillations which was estimated to vary from 15 to 40 stimuli presentations. In both experiments, Δ was distributed within the interval of [0, 40] (see Figure 5C). This distribution displays a significantly larger number of rapid switches (< 5 presented cubes) observed during EXP1, whereas medium (5–10 presented cubes) and optimal (11–20 presented cubes) intervals between switches dominated in EXP2. Taking into account that the period of *I*(*i*) oscillations occurred in the same range, we can conclude that the switching regime in EXP2 mostly satisfied the criterium described above, and therefore led to an increase in operator's performance.

Figures 5D,E illustrate the results of the connectivity analysis in coupled pairs during EXP1 and EXP2. We estimated directions and time lags in coupling between participants by calculating the Recurrence-based Measure of Dependence (RMD) (Goswami et al., 2013) (see detailed description in section 2.) using time series *I*_1_(*i*) and *I*_2_(*i*) recorded during EXP1 and EXP2. The time lag τ was introduced by a relevant shifting of one of the time series relative to the other by τ units. We found that during EXP1 (Figure 5D) the participants mostly performed their tasks independently or rarely influencing each other at short lags (about 10 image presentations). Indeed, a large number of fast spontaneous switches between high and low complexity tasks could not determine the effective interaction between participants. On the contrary, during EXP2 (Figure 5E) the delayed coupling between participants caused the establishment of unidirectional dependence of the assistant on the leader, that was reproduced for all pairs of participants. Notably, the most significant time lag of this dependence, observed in the most of pairs and corresponded to approximately 20 image presentations, is associated with natural frequency of individual brain response oscillations.

## 4. Discussion

The possibility to estimate and control the human psychophysiological condition, and the ability to accomplish mental tasks via BCI is of great fundamental and practical interest (Borghini et al., 2017). In this context, the estimation and control of such human factor as alertness is very important (Lim et al., 2012; Liu et al., 2013; Ko et al., 2017). Alertness manifests itself as an active attentional state marked by high sensory awareness to be watchful and ready for any potential danger or emergency, or fast to respond to it. As it was shown in neurophysiological studies, a person who has to be alerted during his/her work, such as air traffic controllers or pilots, often has a trouble to maintain alertness. In some situations, people use drugs in order to increase attention, but a safer and more effective way is to use BCI to improve the human ability to maintain alertness. Such BCIs can give rise to the development of training systems, in particular, for children with attention deficit hyperactivity disorder (Lim et al., 2012; Hillard et al., 2013), as well as assistant systems which allow to control the attention during long-lasting job tasks. Moreover, they can be used for the development of BCI for completely paralyzed people (Hill and Schölkopf, 2012; Jin et al., 2017).

The BCIs which are aimed at increasing human brain abilities are known as passive BCIs. These BCIs use biomarkers extracted from brain signals to improve human's cognitive performance with no aim of voluntary control of the system (Zander and Kothe, 2011). The main goal of such systems is to enhance cognitive performance of healthy humans and to recover mild cognitive impairment. In this context, traditional definition of BCI is more suitable for active BCIs. They imply that operator voluntarily generates specific patterns of brain activity which can be automatically detected in real time and translated into commands for controlling a technical device by thought. Active BCIs mostly aim to help paralyzed people to communicate and interact with external environment (Wolpaw et al., 2002).

Passive BCIs are able to estimate degree of alertness in real time based on changes in the time-frequency structure of human EEG and provide possibility for alertness control by biological feedback (Maksimenko et al., 2017b). At the same time, the ability of such BCIs to immediately improve human alertness is not reported in literature. The recent review on the methods for the improvement of cognitive performance (Taya et al., 2015) states that brain abilities can be enhanced by prolonged and systematic cognitive training. In this paper, we propose an alternative approach. We demonstrate that human alertness can be immediately improved via human-to-human interaction. In particular, we shown, for the first time to the best of our knowledge, that human performance to perform a cognitive load can be increased using BBI which monitors working ability of every person in the group and distributes the load among all participants according to their individual ability, in order to perform a common task more efficiently. This became possible due to specific EEG hallmarks associated with degree of alertness.

Having analyzed the mean degree of alertness, we have found that in each pair of participants, the subject who initially demonstrated higher degree of alertness in the experiments without any interaction, exhibited increasing alertness in the experiment with human-to-human interaction due to the assistance of his/her partner. It was surprising that the increasing alertness was only observed in the experiment, where the task complexity was changed as soon as the difference in the degree of alertness between the partners exceeded 10%. We have shown that this effect is caused by the oscillatory behavior of the degree of alertness, determined by the average period of cognitive recovery after mental fatigue. In this respect, the effective interval between switches of the task complexity should coincide with this brain rhythm.

The obtained results demonstrate the principal difference between human-machine and human-to-human interactions. While in the former case, the machine performs a part of a cognitive or a physical human load, in the latter case, the machine is replaced by another human, so that a feedback is required to compare the subjects' brain states in order to decide who is more suitable for the action. This is especially important for systems aimed at increasing human cognitive performance. Since brain ability to consciously perceive and process information is limited (Marois and Ivanoff, 2005), the human exhibits a state of mental fatigue manifested as brain inability to complete the mental task which requires a high level of sustained attention in the absence of discernible cognitive failure (Chaudhuri and Behan, 2000).

Human-to-human interaction can also be implemented via special brain-to-brain interfaces which along with active and passive BCIs, have become a hot topic in neuroscience, physics and IT-technologies. Recently, the possibility of human-to-human interaction via BBI was performed in a way, where motor information registered in the cortical region was transmitted to the motor cortex region of another subject with the help of brain stimulation. Such possibility was first demonstrated by Pais-Vieira et al. (2013) in rats. One year later, human-to-human interaction was considered by Rao et al. (2014), who proposed a noninvasive interface which combined EEG with transcranial magnetic stimulation (TMS) for delivering information to brain. Their BBI detected motor imagery in EEG signals recorded from one subject (“sender”) and transmitted this information over Internet to the motor cortex region of another subject (“receiver”).

The brain-to-brain interaction was also implemented in the form of human-animal interaction (Yoo et al., 2013), where the human volunteer's intention was translated to stimulate a rat's brain motor area responsible for the tail movement. Another type of BBI was demonstrated by Mashat et al. (2017) in a closed-loop form, where the intention signal from a sender was recognized using EEG and sent out to trigger TMS of a receiver to induce hand motion; meanwhile, TMS resulted in a significant change in the motor evoked potentials recorded by electromyography of the receiver's arm, which triggered functional electrical stimulation applied to the sender's arm and generated hand motion.

Although these studies provided the experimental evidence of the information transmission between brains, they did not demonstrate the possibility to improve the performance of a sender and a receiver. The control command was translated to the receiver's brain in any case, not considering its willingness to perform an action. In other words, previously proposed systems did not take into account brain states of interacting people. Instead, our BBI analyses and compares human brain states, as a core feature of the human-to-human interaction, to improve human performance in tasks which require sustained attention, in particular, in classification of consecutively presented ambiguous images. At the same time, possible applications of proposed BBI are widespread. The distribution of cognitive or physical load unequally over all participants depending on their current psychophysiological conditions is a very efficient way to improve the working performance of the group of people.

## 5. Conclusion

The presented results contributed in the multidisciplinary field of science, especially, in physics and brain-to-brain interface development. From a physical point of view, we have considered interacting human brains as a mutually coupled dynamical system and revealed nonlinear phenomena caused by particular features of electrical brain activity. In particular, we have found that the brain activity, while performing a cognitive load, oscillates in time with a certain mean period which can be related to periodic cognitive recovery after mental fatigue caused by a difficult task. The modulation of the cognitive load complexity with a frequency close to this “natural” frequency of cognitive recovery allows maintaining a high brain performance for a prolonged time. Moreover, such a modulation increases the amplitude of the brain response, similar to a resonant behavior of a dynamical system subjected to an external periodic forcing. This principle underlies the proposed BBI aimed to increase human performance due to brain-to-brain interaction.

The obtained results can be a starting point for the development of systems for neural-based communication between humans which allow to “feel” conditions of the partner and make the human-to-human interaction more efficient. This would be very helpful for a group of people subjected to a common task which requires sustained attention and alertness. The BBI could help such people to have effective interactions by estimating and monitoring physical conditions of each team member, in particular, degree of alertness, in order to distribute workloads among all participants according to their current physiological status. Starting from the case of two interacting persons, this system can be extended to the large network of people working on a common project.

## Author Contributions

VAM, AEH, ANP, and JK conceived the study. VVG, AL, and AER performed experiments. VAM and VON implemented data processing algorithm. VVM and NSF analyzed the data. VAM, AEH and ANP wrote the manuscript.

### Conflict of Interest Statement

The authors declare that the research was conducted in the absence of any commercial or financial relationships that could be construed as a potential conflict of interest.
